# Euthanasia and assisted suicide (EAS) in psychiatric patients

**DOI:** 10.1192/j.eurpsy.2023.404

**Published:** 2023-07-19

**Authors:** P. Pedro Felgueiras, R. Silva

**Affiliations:** Psychiatry department, Vila Nova de Gaia Hospital Center, Vila Nova de Gaia, Portugal

## Abstract

**Introduction:**

A legal definition for EAS describes this procedure as “intentionally terminating life by someone other than the person concerned, at the latter’s request”. The number of requests for EAS has been progressively increasing in countries where this procedure is allowed, including concerning psychiatric patients (2% of all requests). EAS for reasons of unbearable suffering raises ethical concerns due to lack of criteria for psychiatric patients.

**Objectives:**

To discuss the avaliable data about EAS and its controversial value in psychiatric patients.

**Methods:**

Non-systematic review of literature on current knowledge about EAS, particularly in patients with mental disorder.

**Results:**

In terms of sociodemographic and clinical characteristics, these patients were mostly women, with at least two psychiatric conditions; the main diagnosis is a (treatment-resistant) mood disorder, with some medical comorbidity. Psychological suffering was the main motivation, in patients with severe symptomatology associated with psychiatric and physical conditions (26% reported both psychological and physical suffering). These patients tend to be empowered and value self-determination. There is to highlight a high percentage of patients still alive after a not granted pEAS request (69%) and a high rate of pEAS requests withdrawals (37%).

**Conclusions:**

Suicide prevention remains a priority in terms of public health. Thus, there is a need to ensure that EAS isn`t a way to increase suicide mortality by giving access to lethal methods to suicidal patients. In some cases, EAS request has a paradoxical value to regain control of life and it`s related to the transient nature of unbearable mental suffering.

The actual process provides a continued recovery-oriented care in parallel with the EAS evaluation, and a thorough evaluation which requires a multi-expert panel with the envolvement of mental health professionals. Ethical concerns remains about its paradox: unbearable psychological suffering is a target for suicide prevention and also a required criterion for EAS.

**Disclosure of Interest:**

None Declared

